# Effects of Hypericum Perforatum, in a rodent model of periodontitis

**DOI:** 10.1186/1472-6882-10-73

**Published:** 2010-11-23

**Authors:** Irene Paterniti, Enrico Briguglio, Emanuela Mazzon, Maria Galuppo, Giacomo Oteri, Giancarlo Cordasco, Salvatore Cuzzocrea

**Affiliations:** 1 Department of Clinical and Experimental Medicine and Pharmacology, School of Medicine, University of Messina, Italy; 2 Istituto Policattedra di Odontostomatologia, University of Messina, Italy; 3 IRCCS Centro Neurolesi "Bonino-Pulejo", Messina, Italy

## Abstract

**Background:**

*Hypericum perforatum *is a medicinal plant species containing many polyphenolic compounds, namely flavonoids and phenolic acids. In this study we evaluate the effect of *Hypericum perforatum *in animal model of periodontitis.

**Methods:**

Periodontitis was induced in adult male Sprague-Dawley rats by placing a nylon thread ligature around the lower 1st molars. Hypericum perforatum was administered at the dose of 2 mg/kg os, daily for eight days. At day 8, the gingivomucosal tissue encircling the mandibular first molar was removed.

**Results:**

Periodontitis in rats resulted in an inflammatory process characterized by edema, neutrophil infiltration and cytokine production that was followed by the recruitment of other inflammatory cells, production of a range of inflammatory mediators such as NF-κB and iNOS expression, the nitration of tyrosine residues and activation of the nuclear enzyme poly (ADP-ribose) polymerase; apoptosis and the degree of gingivomucosal tissues injury. We report here that Hypericum perforatum exerts potent anti-inflammatory effects significantly reducing all of the parameters of inflammation as described above.

**Conclusions:**

Taken together, our results clearly demonstrate that treatment with Hypericum reduces the development of inflammation and tissue injury, events associated with periodontitis.

## Background

Human periodontal diseases are inflammatory disorders that give rise to damage the surrounding cells and connective tissue structures, including alveolar bone causing tooth loss [1]. The periodontal disease is infections that are caused by accumulation of bacteria that colonize the tooth surface at or below the gingival margin. Microbial plaque is recognized as the primary etiological agent for periodontal disease initiation and progression [2]. Generally there is a further enhancement of the inflammatory state as exposure to plaque continues, there is increase fluid exudation and leukocyte migration into the tissues and the gingival crevice [1].

The progression of chronic periodontitis is a continuous process that undergoes periods of acute exacerbation [3]. The established lesion is dominated by plasma cells, that are situated primarily in the coronal connective tissues as well as around vessels, collagen loss continues in both lateral and apical directions as the inflammatory cell infiltrate expands, resulting in collagens extending spaces deeper into the tissues which are then available for leukocyte infiltration [4].

There are two types of established lesion: one remains stable and is not progressing for month or years, the second one becomes more active and converts to a progressive and advanced lesion [1,4]. The severity of periodontitis is characterized by the degree of marginal bone loss, the depth of periodontal pockets, the degree of attachment loss and the number of teeth with furcation development [5]. In recent years, more attention has been focused on the role of reactive oxygen species, lipid peroxidation products and antioxidant systems in the pathology of periodontitis [6].

Recent medical and dental research in this area has been geared towards the prevention of free radical mediated diseases by using specific nutrient antioxidants [7].

Other important candidate factors that may modulate periodontitis, are pro-inflammatory cytokines [such as tumor necrosis factor-a (TNF-a), interleukin (IL)-1β] known to be up-regulated early in the course of periodontitis. In addition, recruitment of inflammatory cells from the circulation is an important process to augment the inflammatory response [8]. Pro-inflammatory cytokines production also induces the expression of adhesion molecules in the vascular endothelium, and invasion of inflammatory cells into inflamed tissues subsequently occurs. P-selectin, a member of the selectin family of adhesion molecules, and intercellular adhesion molecule-1 (ICAM-1), both of which are expressed at the surface of the vascular endothelium, are involved in this process. Various mediators contribute to the up-regulation of endothelial cell and leukocyte-adhesion molecules in inflammation.

*Hypericum perforatum *L. (Hypericaceae), popularly called St. John's wort, is an herbaceous perennial plant belonging to the family Clusiaceae, which is used in popular medicine and phytotherapy for its well documented antiseptic and antidepressant effects. Moreover, it has been proposed to have antibacterial and antiviral effects and to exert anti-inflammatory and analgesic activity. Hypericum perforatum extract contains flavonoids and phenolic acids, which demonstrated a free radical scavenging activity. The Hypericum perforatum extract exerts very efficient anti-inflammatory effects in animal model of acute inflammation [9].

Hypericum extract, is a very efficacious antidepressant medication with a potential antioxidant activity, was therefore conjectured to be useful in the treatment of pathological situation in which ROS play an important role such as acute inflammation. Thus, the aim of the present study was to evaluate the effects of Hypericum perforatum extract in a rat experimental model of periodontitis.

To gain a better insight into the mechanism(s) of action, we have evaluated the following end points of the inflammatory process: (1) histological damage, (2) bone loss (radiography), (3) cytokine expression (4) nitrotyrosine, and inducible nitric oxide synthase (iNOS) expression and (5) apoptosis.

## Methods

### Hypericum perforatum extract

*Hypericum perforatum *methanolic extract was a kind gift of Indena (Milano, Italy), and it was defined by the producer as containing 0,34% of hypericin, 4,1% of hyperforin, 5% of flavonoid (rutin, kaempferol, luteolin, myrecitin, quercitin, quercitrin, isoquercitrin), 10% tannins and the remaining part is composed of polysaccharides represented by maltodextrins.

### Surgical Procedure

Male Sprague Dawley rats (280-400 gr) were lightly anaesthetized with surgical doses of sodium pentobarbitone (35 mg/kg). Sterile, 2-0 black braided silk thread was placed around the cervix of the lower left first molar and knotted medially as previously described (Di Paola et al., 2004). After the rats had recovered from the anesthetic they were allowed to eat commercial laboratory food (standard rodent chow) and drink tap water ad libitum. Animals and the study protocol were approved by the institutional Animal Care and User committee of the University of Messina.

### Experimental groups

Rats were randomly allocated into the following groups:

*Ligature + vehicle groups*: rats were subjected to ligature placed around the gingival margin of the mandibular of the first molar that induced periodontitis, and animals received vehicle intraperitoneally (i.p.; daily treatment for eight days).

*Ligature + Hypericum perforatum group: *rats were subjected to ligature-induced periodontitis and animals received Hypericum (2 mg/kg orally, daily for eight days).

At 8 days after the ligature-induction of periodontitis the rats (N 10 from each group for each parameter) were sacrificed in order to evaluate the various parameters described below. The right side that is not subject to ligature was used as control.

### Histological examination

For histopatological examination, biopsies of gingival and mucosa tissue from the buccal and lingual aspect of the teeth were taken 8 days after the ligature-induction of periodontitis. The tissue slices were fixed in 10% neutral-buffered formaldehyde for 5 days, embedded in paraffin, and sectioned. The sections, oriented longitudinally from the teeth crowns, were stained with Masson's trichrome stain to visualize collagen distribution in the gingival and mucosa tissue. The total number of infiltrating leukocytes (e.g., neutrophils and mononuclear cells) in cortical interstitial spaces from gingival and mucosa tissues were assessed quantitatively by counting the number of polymorphonuclear cells in 20 high power fields.

### Radiography

Mandibles were placed on a radiographic box at a distance of 90 cm from the x-ray source. Radiographic analysis of normal and legated mandibles was performed by x-ray machine (Philips × 12, Germany) with a 40 kw exposure for 0, 01 sec. A radiographic examination of at eight day after ligature placement revealed bone matrix resorption in the lower first left after legation as previously described.

### Measurement of vascular permeability by Evans blue extravasations

Vascular permeability was determinate as previously described [10]. Briefly, animals received Evans blue (2.5% dissolved in physiological saline, at a dose of 50 mg/kg) via a femoral venous catheter. Extravasated Evans blue in the excised gingivomucosal tissue samples was extracted with 1 ml formamide for 48 h at room temperature for spectrophotometric determination at 620 nm and expressed as μg/g gingivomucosal tissue [10].

### Measurement of alveolar bone loss

The distance from the cementoenamel junction of first lower molars to the alveolar crest was measured with a modification of the method of [11]. Recordings were made along the median axis of the lingual surface of the mesial and mediolingual roots of the lower first left and right molars as previously described [6]. These measurements were performed by an independent investigator who was unaware of the treatment regimens. The alveolar bone loss induced by the ligature was expressed as a difference between the left and the right side.

### Myeloperoxidase activity

Myeloperoxidase activity, an indicator of polymorphonuclear leukocyte (PMN) accumulation, was determined in gingivomucosal tissue, as previously described [12]. Myeloperoxidase activity was defined as the quantity of enzyme degrading 1 μmol/min of peroxide at 37 °C and was expressed in milliunits/g of wet tissue.

### Immunohistochemical localization of nitrotyrosine, iNOS, P-selectin, ICAM and IL-1

At the end of the experiment, the tissues were fixed in 10% (w/v) PBS-buffered formaldehyde and 8 μm sections were prepared from paraffin embedded tissues. After deparaffinization, endogenous peroxidase was quenched with 0.3% (v/v) hydrogen peroxide in 60% (v/v) methanol for 30 min. The sections were permeablized with 0.1% (w/v) Triton X-100 in PBS for 20 min. Non-specific adsorption was minimised by incubating the section in 2% (v/v) normal goat serum in PBS for 20 min. Endogenous biotin or avidin binding sites were blocked by sequential incubation for 15 min with biotin and avidin (DBA, Milan, Italy), respectively. Sections were incubated overnight with 1) purified goat polyclonal antibody directed towards P-selectin which reacts with mice; or 2) with purified hamster anti-mouse ICAM-1 (CD54) (1:500 in PBS, w/v) (DBA, Milan, Italy) or 3) with anti-nitrotyrosine rabbit polyclonal antibody (1:500 in PBS, v/v) or 4) with anti-iNOS antibody (1:500 in PBS, v/v) or 5) with anti-IL-1β polyclonal antibody (Santa Cruz Biotechnology, 1:500 in PBS, v/v). Sections were washed with PBS, and incubated with secondary antibody. Specific labelling was detected with a biotin-conjugated goat anti-rabbit IgG and avidin-biotin peroxidase complex (DBA, Milan, Italy). In order to confirm that the immunoreaction for the nitrotyrosine was specific some sections were also incubated with the primary antibody (anti-nitrotyrosine) in the presence of excess nitrotyrosine (10 mM) to verify the binding specificity. To verify the binding specificity for ICAM-1, P-selectin, iNOS and IL-1β, some sections were also incubated with only the primary antibody (no secondary) or with only the secondary antibody (no primary). In these situations no positive staining was found in the sections indicating that the immunoreaction was positive in all the experiments carried out. Immunocytochemistry photographs (n 5 photos from each samples collected from all rats in each experimental group) were assessed by densitometric analysis by using Optilab Graftek software on a Macintosh personal computer.

### Western blot analysis for IκB-α, NF-κB p65, Bax, Bcl-2, iNOS

Cytosolic and nuclear extracts were prepared as previously described [13] with slight modifications. Briefly, tissue samples from ligature-operated rats were suspended in extraction Buffer A containing 0.2 mM phenylmethylsulphonyl fluoride (PMSF), 0, 15 μM pepstatin A, 20 μM leupeptin, 1 mM sodium orthovanadate, homogenized at the highest setting for 2 min, and centrifuged at 1,000 × g for 10 min at 4 °C. Supernatants represented the cytosolic fraction. The pellets, were re-suspended in Buffer B containing 1% Triton X-100, 150 mM NaCl, 10 mM TRIS-HCl pH 7.4, 1 mM ethylene glycol-bis(beta-aminoethyl ether)-N, N, N', N'-tetraacetic acid (EGTA), 1 mM ethylene diamine-tetra-acetic acid (EDTA), 0,2 mM PMSF, 20 μM leupeptin, 0,2 mM sodium orthovanadate. After centrifugation 30 min at 15,000 × g at 4 °C, the supernatants containing the nuclear protein were stored at -80 °C for further analysis. Protein concentration in homogenate was determined by Bio-Rad Protein Assay (BioRad, Richmond CA) and 50 mg of cytosol and nuclear extract from each sample was analyzed. The levels of IkB-α, iNOS, Bax, and Bcl-2 were quantified in cytosolic fractions from tissue samples, while NF-κB p65 levels were quantified in nuclear fractions. The membranes of nitrocellulose were blocked with 1× PBS, 5% (w/v) non fat dried milk (PM) for 40 min at room temperature and subsequently probed with specific Abs IkB-α (Santa Cruz Biotechnology, 1:1000), or anti-Bax (1:500; Santa Cruz Biotechnology), or anti-Bcl-2 (1:500; Santa Cruz Biotechnology), or anti-iNOS, (1:1000 Signal Transduction) or anti-NF-κB p65 (1:1000; Santa Cruz Biotechnology) in 1× PBS, 5% w/v non fat dried milk, 0.1% Tween-20 (PMT) at 4 °C, overnight. Membranes were incubated with peroxidase-conjugated bovine anti-mouse IgG secondary antibody or peroxidase-conjugated goat anti-rabbit IgG (1:2000, Jackson ImmunoResearch, West Grove, PA) for 1 h at room temperature.

To ascertain that blots were loaded with equal amounts of protein lysates, they were also incubated in the presence of the antibody against β-actin (1:10,000 Sigma-Aldrich Corp.). The relative expression of the protein bands of IκB-α (~37 kDa), iNOS (~130 kDa), NF-kB p65 (65 kDa), Bax (~23 kDa), Bcl-2 (~29 kDa) was quantified by densitometric scanning of the X-ray films with GS-700 Imaging Densitometer (GS-700, Bio-Rad Laboratories, Milan, Italy) and a computer program (Molecular Analyst, IBM).

### Materials

All compounds were obtained from Sigma-Aldrich Company Ltd. (Milan, Italy). All other chemicals were of the highest commercial grade available. All stock solutions were prepared in non-pyrogenic saline (0.9% NaCl; Baxter, Italy, UK).

### Statistical evaluation

All values in the figures and text are expressed as mean +/- standard error (s.e.m.) of the mean of n observations. For the in vivo studies n represents the number of animals studied. In the experiments involving histology or immunohistochemistry, the figures shown are representative of at least three experiments (histological or immunohistochemistry coloration) performed on different experimental days on the tissue sections collected from all the animals in each group. The results were analyzed by one-way ANOVA followed by a Bonferroni post-hoc test for multiple comparisons. A p-value less than 0.05 were considered significant. And individual group means were then compared with Student's unpaired t test. A P-value of less than 0.05 was considered significant.

## Results

### Effect of *Hypericum *on tissue damage and radiographic evaluation of ligature

**Figure 1:**
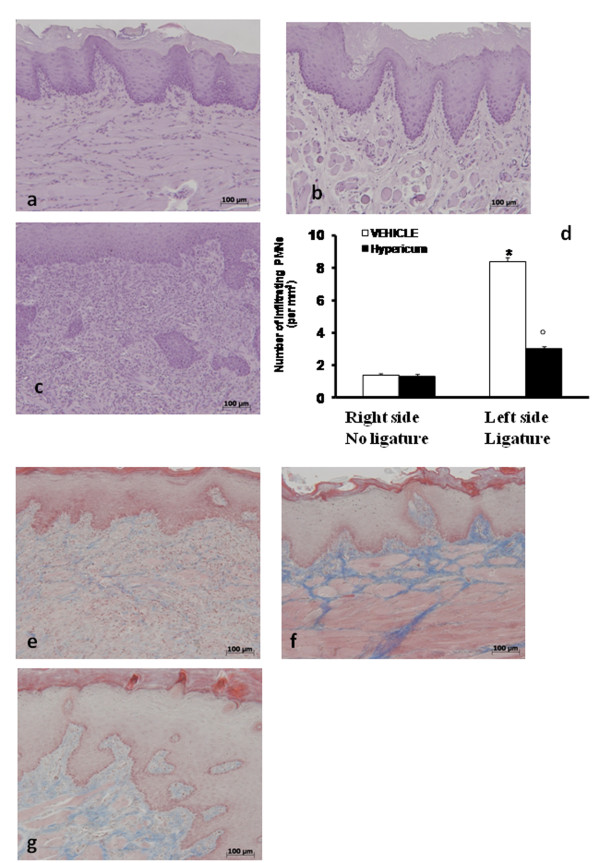
**Inflammatory cells infiltration and edema were observed in gingivomucosal section from ligature-treated rats (b) when compared with gingivomucosal tissue section taken from controlateral side (a)**. Significantly less edema and inflammatory cell infiltration was observed in gingivomucosal sections from ligature-treated rats which had been treated with Hypericum (c). The total number of infiltrating leukocytes (e.g., neutrophil and mononuclear cells) in gingivomucosal tissue was assessed quantitatively by counting the number of polymorphonuclear cell in 20 high-power fields (d). Moreover Masson's trichrome stain was negative in gingivomucosal tissue sections taken from the contralateral side from vehicle when compared with gingivomucosal tissues sections of ligature-operated rats (e, f.). *Hypericum *treatment reduced the increase of collagen (Fig g) Figures are representative of at least 3 experiments performed on different experimental days. The tissue sections, orientated longitudinally from the teeth crown, were stained with trichrome stain. Data represent the mean ± S.E.M. for 20 counts obtained from the gingivomucosal tissue of each treatment group. * < P0.01 vs. non-ligated; °P < 0.01 vs. ligated.

When compared with gingivomucosal tissue sections taken from the contralateral side from vehicle (Figure 1a) and *Hypericum *(Figure 1c.)-treated rats, histological examination of gingivomucosal tissues sections of ligature-operated rats showed edema, tissue injury as well as infiltration of the tissue with inflammatory cells (Figure 1b). *Hypericum *treatment reduced the degree of gingivomucosal tissues injury (Figure 1c.). Quantification of infiltrating polymorphonuclear cell into gingivomucosal tissue showed that there were only a minimal number of polymorphonuclear cells in tissue from the contra lateral side (Figure 1d.). However, a large number of infiltrating polymorphonuclear cell were observed in the gingivomucosal tissue of ligated rats (Figure 1d.). Hypericum administration significantly reduced the numbers of polymorphonuclear cell infiltrating into gingivomucosal tissue (Figure 1d.) Moreover Masson's trichrome stain, which is used to monitor the increase of collagen fiber, was negative in gingivomucosal tissue sections taken from the contralateral side from vehicle when compared with gingivomucosal tissues sections of ligature-operated rats (Figure 1e, f). *Hypericum *treatment reduced the increase of collagen (Figure 1g). Data represent the mean ± S.E.M. for 20 counts obtained from the gingivomucosal tissue of each treatment group * < P0.01 vs. non-ligated. °P < 0.01 vs. ligated.

### Effect of *Hypericum *on alveolar bone loss

**Figure 2:**
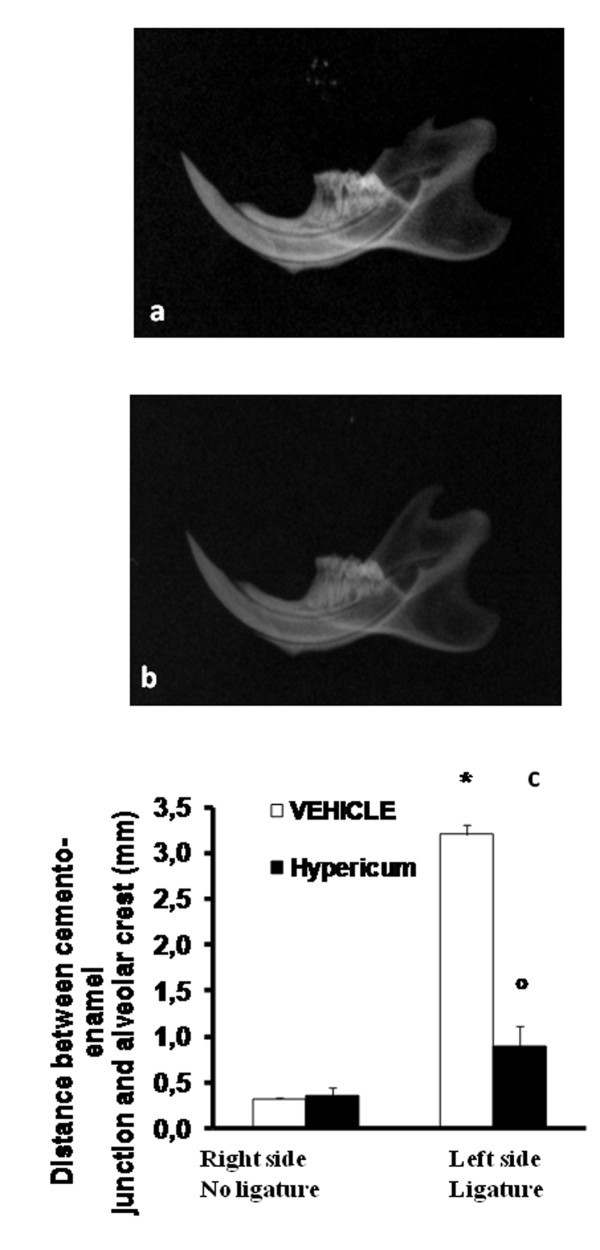
**The alveolar bone from ligated (8 days) rats demonstrated alveolar bone resorption (a)**. Hypericum treatment suppressed alveolar pathology in the rat alveolar bone (b). A significant increase in the distance between cemento-enamel injunction and alveolar crest at mediolingulal root of the first molar was observed in ligature-treated rats (c). Hypericum treatment significantly reduced the increase in the distance between cemento-enamel injunction and alveolar crest (c). Radiographic figure is representative of at least 3 experiments performed on different experimental days. Data represent the data from 20 counts obtained from the gingivomucosal tissue of each treatment group. *P < 0.01 vs. non-ligated; °P < 0.01 vs. ligated.

A radiographic examination of the mandibles, at day 8 after ligature placement, revealed bone matrix resorption in the lower left first molar region after ligation (Figure 2a.). There was no evidence of pathology in theright first molar (data not shown). *Hypericum *markedly reduced the degree of bone resorption in the lower left first molar region after ligation (Figure 2b). A significant alveolar bone loss between the lower first left molar and the right first molars induced by the left side ligature was observed in vehicle-treated rats. Hypericum treatment resulted in a significant inhibition of alveolar bone loss after ligation (Figure 2c). In addition, a significant alveolar bone loss, between the lower first left and the right first molars induced by the left side ligature, was observed in vehicle treated rats (Figure 2c.). Hypericum treatment resulted in a significant inhibition of alveolar bone loss after ligation (Figure 2c). Data represent the mean ± S.E.M. for 20 counts obtained from the gingivomucosal tissue of each treatment group * < P0.01 vs. non-ligated. °P < 0.01 vs. ligated.

### Effect of Hypericum on IκB-α degradation and NF-κB p65 activation

We evaluated IκB-α degradation and nuclear NF-κB p65 by Western Blot analysis to investigate the cellular mechanisms by which treatment with Hypericum may attenuate the development of periodontitis.

**Figure 3:**
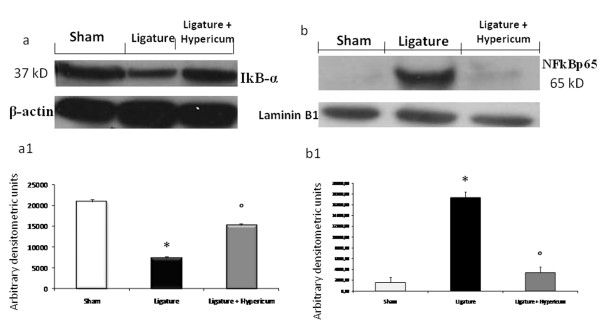
**We evaluated IκB-α degradation and nuclear NF-κB p65 by Western Blot analysis**. A basal level of IκB-α was detected in the gingivomucosal tissue sections taken from the contralateral side (a, a1). IκB-α levels were substantially reduced (a, a1.) in the gingivomucosal tissues from ligature operated rats. Hypericum treatment prevented IκB-α degradation, (3a, a1). Periodontitis caused a significant increase in the NF-kB p65 levels in the gingivomucosal tissues from operated rats (b, b1). Hypericum treatment significantly prevented the periodontitis-mediated NF-kB p65 expression (b, b1).

A basal level of IκB-α was detected in the gingivomucosal tissue sections taken from the contralateral side from vehicle (Figure 3a, a1), whereas 8 days following ligation IκB-α levels were substantially reduced (Figure 3a, a1) in the gingivomucosal tissues from ligature operated rats. Hypericum treatment prevented IκB-α degradation, the IκB-α levels observed in these animals were similar to those of of gingivomucosal tissues from the contralateral side (Figure 3a, a1).

In addition, periodontitis caused a significant increase in the NF-kB p65 levels in the nuclear fractions from of gingivomucosal tissues from operated rats (Figure 3b, b1) compared to the of gingivomucosal tissues from the contralateral side (Figure 3b, b1). Hypericum treatment significantly prevented the periodontitis-mediated NF-kB p65 expression (Figure 3b, b1). Data represent the mean ± S.E.M. for 20 counts obtained from the gingivomucosal tissue of each treatment group * < P0.01 vs. non-ligated. °P < 0.01 vs. ligated.

### Effects of Hypericum on plasma extravasation and neutrophils infiltration in periodontitis

**Figure 4:**
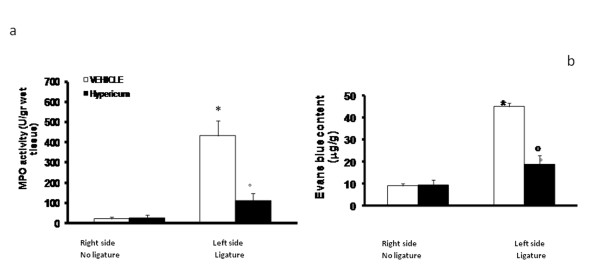
**Evans blue content (b) and Myeloperoxidase activity (a) in gingivomucosal tissue was significantly increased by ligature compared to the contralateral side**. Hypericum significantly reduced myeloperoxidase activity levels and Evans blue content (a, b respectively). Densitometry data are expressed as % of total tissue area. Data are means of mean ± s.e.m. from N = 10 rats for each group. *P < 0.01 vs. non-ligated. ° < b0.01 vs. ligated.

Before the measurement of Evans blue extravasation, mean arterial pressure of vehicle-treated and Hypericum-treated animals was recorded. In agreement with previous studies [14], Hypericum treatment did not affect mean arterial blood pressure (vehicle-treated: 128+6 mm Hg; N = 10 and Hypericum treated: 125+7 mm Hg; N = 10). Ligation significantly increased Evans blue extravasation in gingivomucosal tissue compared to the contralateral side (Figure 4b). Hypericum treatment prevented this increase in Evans blue extravasation, but did not change the Evans blue content of the contralateral side (Figure 4b.). Myeloperoxidase activity was significantly elevated at eight days after the ligature (Figure 4a.) and Hypericum-treatment significantly reduced these levels (Fig). No significant changes of myeloperoxidase activity were observed in the gingivomucosal tissues from the contra lateral side (Figure 4a). Data represent the mean ± S.E.M. for 20 counts obtained from the gingivomucosal tissue of each treatment group * < P0.01 vs. non-ligated. °P < 0.01 vs. ligated.

### Hypericum modulates cytokines expression

**Figure 5:**
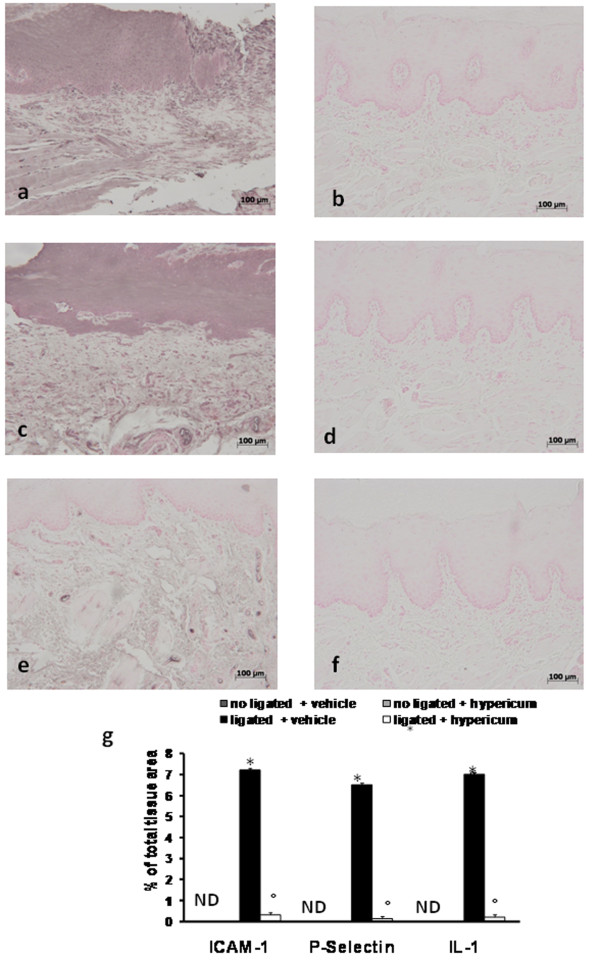
**Positive staining for IL-1β (a, see densitometry analysis g) was observed in gingivomucosal tissue after ligature mainly localized in the epidermis and in inflammatory cells in derma In gingivomucosal tissue of Hypericum-treated rats no positive staining was observed for IL-1β (b, see densitometry analysis g)**. Moreover 8 days following ligation, positive staining for ICAM-1 and P-selectin, were found in the gingivomucosal tissues from ligature operated rats (c and e respectively, see densitometry analysis g). In contrast, a positive staining for ICAM-1 and P-selectin was significantly attenuated by the treatment with Hypericum (d and f respectively, see densitometry analysis g). Figure is representative of at least 3 experiments performed on different experimental days. Densitometry data are expressed as % of total tissue area. Data are means of mean ± s.e.m. from N = 10 rats for each group. *P < 0.01 vs. non-ligated. ° < b0.01 vs. ligated.

To test whether Hypericum modulates the inflammatory process through the regulation of secretion of pro-inflammatory cytokines, we analyzed by immunohistochemical analysis levels of IL-1β. Immunohistochemical analysis of gingivomucosal tissues from the contralateral side did not reveal any immunoreactivity for IL-1β (data not shown). In contrast, 8 days following ligation, positive staining for IL-1β were found in the gingivomucosal tissues from ligature operated rats (Figure 5a). Hypericum treatment significantly reduced the degree of positive staining for these pro-inflammatory cytokines IL-1β (Figure 5b). Data represent the mean ± S.E.M. for 20 counts obtained from the gingivomucosal tissue of each treatment group * < P0.01 vs. non-ligated. °P < 0.01 vs. ligated

### Effects of Hypericum on the expression of adhesion molecules (ICAM-1, P-selectin)

Immunohistochemical analysis of gingivomucosal tissues from the contralateral side did not reveal any immunoreactivity for ICAM-1 and P-selectin (data not shown). In contrast, 8 days following ligation, positive staining for ICAM-1 and P-selectin, were found in the gingivomucosal tissues from ligature operated rats (Figure 5c and 5e respectively), mainly localized in the inflammatory cells in derma and around the vessels respectively. In contrast, a positive staining for ICAM-1 and P-selectin was significantly attenuated by the treatment with Hypericum (Figure 5d and 5f respectively). Data represent the mean ± S.E.M. for 20 counts obtained from the gingivomucosal tissue of each treatment group * < P0.01 vs. non-ligated. °P < 0.01 vs. ligated.

### Effects of Hypericum on iNOS expression and Nitrotyrosine formation in Periodontitis

**Figure 6:**
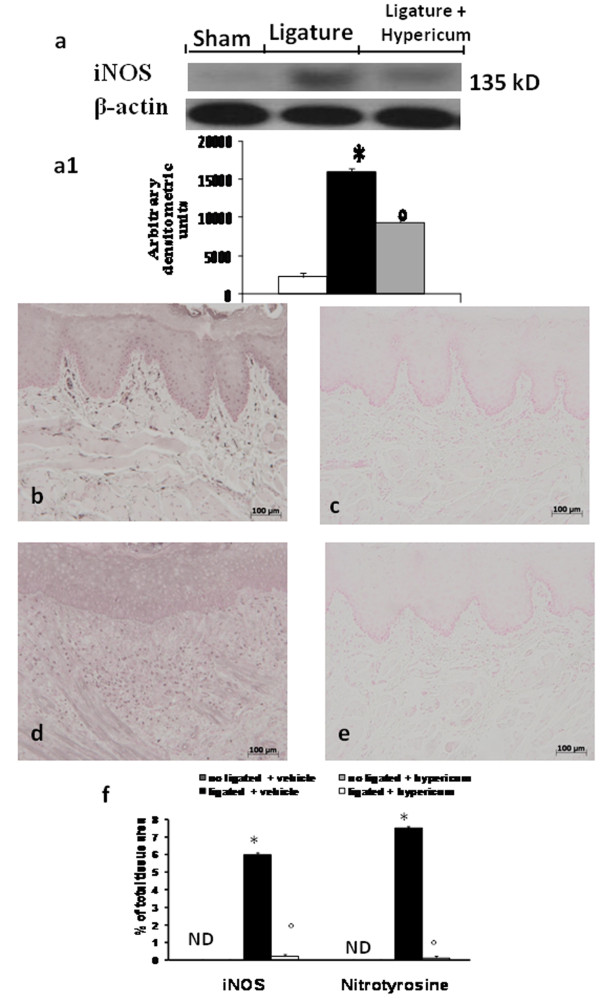
**A significant increase in iNOS expression, assayed by Western blot analysis, was detected in the tissue from ligature-treated rats (a, a1)**. The treatment with Hypericum significantly reduced iNOS expression in the gingivomucosal tissues (a, a1). Moreover positive staining for iNOS (b see densitometry analysis f), and nitrotyrosine (d see densitometry analysis f) was observed in gingivomucosal tissue after ligature. In gingivomucosal tissue of Hypericum treated rats no positive staining was observed for iNOS (c see densitometry analysis f), nitrotyrosine (e, see densitometry analysis f). Figure is representative of at least 3 experiments performed on different experimental days. Densitometry data are expressed as % of total tissue area. Data are means of mean ± s.e.m. from N = 10 rats for each group. *P < 0.01 vs. non-ligated. ° < b0.01 vs. ligated.

Sections of gingivomucosal tissue from the contralateral side did not reveal any immunoreactivity for iNOS and nitrotyrosine, within the normal architecture (data not shown). At 8 days following ligation, positive staining for iNOS (Figure 6b) and nitrotyrosine (Figure 6d), was found in the gingivomucosal tissues from ligature-operated rats. Hypericum treatment abolished the staining for iNOS and nitrotyrosine (Figure 6c and 6e respectively).

Moreover levels of iNOS in gingivomucosal tissues were also evaluated by Western Blot analysis. iNOS levels were substantially increased in the gingivomucosal tissues of saline-treated rats (Figure 6a, a1). In contrast Hypericum treatment prevented the periodontitis-mediated iNOS expression (Figure 6a, a1). Data represent the mean ± S.E.M. for 20 counts obtained from the gingivomucosal tissue of each treatment group * < P0.01 vs. non-ligated. °P < 0.01 vs. ligated.

### Western blot analysis and immunohistochemistry for Bax and Bcl-2

**Figure 7:**
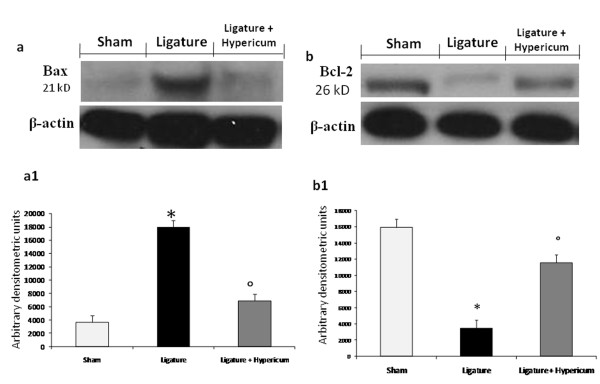
**Western blot analysis was performed on gingivomucosal tissue collected 8 days after injury**. Basal level of Bax was present in the tissue from-sham operated rats (a, a1). Bax band is more evident in the tissue from ligature treated rats (a, a1). The Bax band disappeared in the tissue from ligated rats that received Hypericum (a, a1). Moreover a basal level of Bcl-2 was present in the tissue from sham-operated rats (b, b1). The Bcl-2 band disappeared in the tissue from rats subjected to ligation (b, b1). The Bcl-2 band is more evident in the tissue from ligated rats that received Hypericum (b, b1). The immunoblot in (a and b) represents one tissue of the 5-6 analyzed. The results in (a1 and b1) are expressed as mean 7 s.e.m. from 5-6 tissues. *P < 0.01 vs non-ligated; °P < 0.01 vs ligated.

The appearance of Bax and Bcl-2 in homogenates of gingivomucosal tissues was investigated by Western blot analysis after ligature. A basal level of Bax was detectable in the homogenized gingivomucosal tissues from sham operated animals (Figure 7a, a1). Bax levels were substantially increased in the gingivomucosal tissues of saline-treated rats (Figure 7a, a1). In contrast Hypericum treatment prevented the periodontitis-mediated Bax expression (Figure 7a, a1). A low basal level of Bcl-2 expression was detected in gingivomucosal homogenates from tissue of sham-operated rats (Figure 7b, b1). The expression of Bcl-2 was significantly diminished in whole extracts obtained from gingivomucosal tissues of vehicle-treated rats after ligature (Figure 7b, b1). Treatment of rats with Hypericum significantly reduced the ligature-induced inhibition of Bcl-2 expression (Figure 7b, b1). Data represent the mean ± S.E.M. for 20 counts obtained from the gingivomucosal tissue of each treatment group * < P0.01 vs. non-ligated. °P < 0.01 vs. ligated.

## Discussion

The inability to examine initiation and progression of periodontal disease and to assess certain therapies in humans has led to a great interest in the use of animal models in periodontal research.

Rats and mice have been used for the study of periodontal disease [6,15]. Clinically healthy gingiva can be established and maintained in experimental animals, and gingivitis as well as periodontitis occurs in these animals. It is possible to induce experimental periodontitis by placement of peridental silk ligatures or orthodontic elastics as well as by surgical removal of alveolar bone. Although the most appropriate model for studies of periodontal disease pathogenesis in experimental primates appears to involve the application of silk ligatures, some difficulties may occur in establishing periodontal tissue breakdown by using this model.

Periodontitis, a chronic inflammatory disease of periodontal, supports the protection against local microbial attack, this inflammatory reaction may also damage the surrounding cells and connective tissue structures including alveolar bone causing tooth loss [1].In the present study a well established rat model of acute periodontitis was utilized, which involves placing a ligature around the cervix of the mandibular first molar tooth, and a similar model has previously been used in several species.

In this study we focused our attention on a potential anti-inflammatory activity of Hypericum perforatum for treatment of periodontal disease. It has been known that Hypericum perforatum is an herbaceous plant that has been used as a medicinal plant for centuries to fight against infections and for the treatment of respiratory diseases, peptic ulcers and skin wounds. The traditional use of St. John's wort against infections is supported by the early reported antibacterial properties of its extracts. In the early seventies, a Russian scientific group postulated that there should be an antibiotic contained in St. John's wort extracts. They named this postulated antibiotic as hyperforin. It has been confirmed that hyperforin exhibits effective antibacterial activity against multiresistant Staphylococcus aureus and other Gram-positive bacteria.

But what is then the mechanism by which *Hypericum perforatum *exert it's anti-inflammatory action inhibiting periodontitis?

There is a large body of evidence showing that the production of reactive oxygen and nitrogen species plays key roles in a development of chronic inflammatory disease of periodontal [6].

Recent evidence suggests that the activation of NF-κB may also be under the control of oxidant/antioxidant balance [16]. Moreover, various experimental evidence have clearly suggested that NF-κB plays a central role in the regulation of many genes responsible for the generation of mediators or proteins in inflammation [17]. NF-κB is normally sequestered in the cytoplasm, bound to regulatory proteins IκBs. In response to a wide range of stimuli including oxidative stress, infection, hypoxia, extracellular signals, and inflammation, IκB is phosphorylated by the enzyme IκB kinase [18]. The net result is the release of the NF-κB dimer, which is then free to translocate into the nucleus. The exact mechanisms by which Hypericum suppress NF-κB activation in inflammation are not known. We report here that periodontitis caused a significant increase in the nuclear translocation of the subunit p65 in the gingivomucosal tissues from ligature operated rats. Whereas Hypericum treatment significantly reduced the NF-κB translocation. Moreover, we also demonstrate that the Hypericum treatment also inhibited the IκB-α degradation. NF-κB plays a central role in the regulation of many genes responsible for the generation of mediators or proteins in inflammation. These include the genes for TNF-α, IL-1β and iNOS to name but a few [19]. There is good evidence that IL-1β help to propagate the extension of a local or systemic inflammatory process [20]. We have clearly confirmed a significant increase in the IL-1β production in the pleural 8 days following ligation. On the contrary, a significant reduction of IL-1β production was observed in gingivomucosal tissues from ligature operated rats which received Hypericum.

Our study also confirmed earlier findings, that one of the characteristic signs of inflammation, Evans blue extravasation, was higher on the ligated side on the eighth day, than on the opposite side [10]. In addition, we also report in the present study that ligature-induced peridontitis in the rat results in a significant infiltration of inflammatory cells in the gingivomucosal tissues and we also demonstrated that treatment with Hypericum reduces this inflammatory cells infiltration as assessed by myeloperoxidase and with the moderation of the tissue damage as evaluated by histological examination. Neutrophils are recruited into the tissue and can then contribute to tissue destruction by the production of reactive oxygen metabolites that further amplify the inflammatory response by their effects on macrophages and lymphocytes [21]. A possible mechanism by which Hypericum attenuates polymorphonuclear cells infiltration is by down-regulating adhesion molecules ICAM-1 and P-selectin as previous described [22]. These findings are in accordance with those of Berglund and Lindhe [23] who also found a significant increase in inflammatory cell infiltration in inflamed gingiva as compared to a healthy one. Furthermore, we found that enhanced formation of NO by iNOS may contribute to the inflammatory process. Several studies also support the conclusion that NO from iNOS plays an important role in the pathogenesis of periodontitis [6]. The present study demonstrates that Hypericum attenuates the expression of iNOS in periodontal tissue. Thus, the reduction of the expression of iNOS by Hypericum may contribute to the attenuation by this agent of the formation of nitrotyrosine in the periodontal tissues from ligature-treated rats. Increased nitrotyrosine staining is an indication of "increased nitrosative stress".

Apoptosis, or programmed cell death, is a form of physiological cell death [24]. It is increased or decreased in the presence of infection, inflammation or tissue remodeling. Previous studies have suggested that apoptosis is involved in the pathogenesis of inflammatory periodontal disease [25]. As apoptosis is an exceedingly complex process involving a large variety of signaling molecules, we have focused our attention on a few selective major players. From the results, we identified proapoptotic transcriptional changes, including up regulation of proapoptotic Bax and down regulation of antiapoptotic Bcl-2, using a Western blot assay. This is the first study to show that treatment with Hypericum in periodontitis inhibits and prevents the loss of the antiapoptotic pathway and, also, reduces activation of the proapoptotic pathway by an, as yet, unidentified mechanism.

## Conclusion

In conclusion, this study suggests a potential therapeutic application of Hypericum perforatum extracts for treatment of active inflammatory periodontal disease, this study also demonstrated that Hypericum exerts a significant inhibitory effect on plasma extravasation and reduced the degree of bone resorption during periodontitis.

## List of abbreviations used

IL-1β: interleukin 1β; iNOS: inducible nitric oxide syntethase; MPO: myeloperoxidase; i.p.: intraperitoneally; PBS: Phosphate buffered saline; PMN: polymorphonuclear leukocyte; PMSF: phenyl-methyl sulfonyl fluoride; ROS: reactive oxygen species.

## Competing interests

The authors disclose any financial competing interests and also any non-financial competing interests that may cause them embarrassment were they to become public after the publication of the manuscript.

## Authors' contributions

All authors read and approved the final manuscript.

IP has performed experiment and prepared the manuscript; EB has performed experiment; EM has performed the histological and immunohistochemistry analysis; MG has performed the western blot analysis and analyzed the results; GO and GC have planned the study and analyzed the results and SC has planned the study, analyzed the results and prepared the manuscript.

## Pre-publication history

The pre-publication history for this paper can be accessed here:

http://www.biomedcentral.com/1472-6882/10/73/prepub
